# Intravenous vitamin C as micronutrient therapy in patients with sepsis or septic shock: an updated systematic review and meta-analysis of randomized controlled trials

**DOI:** 10.3389/fnut.2026.1905726

**Published:** 2026-09-04

**Authors:** Binrong Cai, Qian Xiao, Yiqin Xia, Xian Yu, Xiaoyan Xian, Lipeng Liu, Yue Dai, Bin He

**Affiliations:** 1 Department of Emergency Medicine, West China Hospital, Sichuan University, Chengdu, China; 2 Department of Emergency Medicine, West China Tianfu Hospital, Sichuan University, Chengdu, Sichuan, China

**Keywords:** meta-analysis, sepsis, septic shock, systematic review, vitamin C

## Abstract

**Objective:**

The adjunctive therapeutic value of vitamin C in sepsis or septic shock patients remains controversial. This study aimed to evaluate the therapeutic efficacy and safety of intravenous administration of vitamin C in sepsis or septic shock patients through meta-analysis.

**Methods:**

Relevant literature was searched in PubMed, Embase, and Scopus databases and other related databases. The primary outcome was 28-day mortality. Secondary outcomes included duration and total dose of vasopressors, change in sequential organ failure assessment (SOFA) scores from baseline to the earliest reported time point between 72 and 96 h (ΔSOFA), hospital length of stay, intensive care unit length of stay, urine output in the 24-h, and incidence of adverse events.

**Results:**

Fourteen randomized controlled trials enrolling 1,958 patients were eligible for assessment. Meta-analysis demonstrated that intravenous vitamin C reduced 28-day mortality in sepsis or septic shock patients compared to the control group (RR 0.70; 95% CI 0.53 to 0.93; *P* = 0.02; *I*^2^ = 52%), based on low quality of evidence, and subgroup analysis showed the benefit was driven by single-center studies, not observed in multicenter trials. Vitamin C shortened the duration of vasopressor use (SMD = −0.42, 95% CI −0.77 to −0.08, *P* = 0.02) and was associated with a greater reduction in SOFA score (MD = −1.04, 95% CI −1.83 to −0.25, *P* = 0.01), but did not reduce vasopressor dose, length of stay, or 24-h urine output. No significant adverse events were reported.

**Conclusion:**

This meta-analysis suggests that intravenous vitamin C might be associated with reduced mortality in sepsis, but the benefit was not confirmed in low-risk-of-bias multicenter trials and was primarily driven by single-center studies. Intravenous vitamin C appeared safe. Given the exploratory nature of these subgroup analyses and the data-driven thresholds used, high-quality, multicenter RCTs are warranted.

**Systematic review registration:**

http://www.crd.york.ac.uk/PROSPERO/view/CRD4204261367808, identifier CRD4204261367808.

## Introduction

Sepsis, a systemic inflammatory response syndrome triggered by infection, represents a critical condition with exceedingly high incidence and mortality rates in intensive care units (ICUs). Sepsis affects approximately 32 million patients globally each year, with fatalities reaching 5.3 million (1). Sepsis is fundamentally characterized by life-threatening organ dysfunction resulting from the host dysregulated response to infection (2). During this process, excessive oxidative stress plays a role in postinjury endothelial repair processes, promoting adaptive responses to hypoxia and bacterial clearance (3). However, massive release of inflammatory mediators and reactive oxygen species (ROS) after infection-induced immune system activation overwhelms antioxidant defenses, leading to cellular damage, microcirculatory dysfunction, and multiple organ failure (3). The management of patients with sepsis or septic shock typically includes a combination treatment regimen comprising of antimicrobials, fluid resuscitation, vasopressors, and mechanical ventilation when appropriate. However, the mortality of septic shock reaches up to nearly 40% (4).

As a potent antioxidant, vitamin C possesses unique redox properties that directly neutralize free radicals and protect cellular membrane integrity. Given the evidence from previous studies, vitamin C exerts potential protective effects by restoring vascular responsiveness to vasoactive substances, improving microcirculatory blood flow, and preserving endothelial function (5–7). Furthermore, vitamin C serves as an essential cofactor for multiple enzymatic reactions, participating in critical physiological processes including collagen synthesis, catecholamine production, and carnitine biosynthesis (8). However, vitamin C as an essential water-soluble nutrient which cannot be synthesized or stored by humans, entirely relies on exogenous supplementation to maintain vitamin C levels. Sepsis patients frequently develop severe vitamin C deficiency due to increased metabolic demands, reduced intake, and excessive losses. Nearly 90% of sepsis patients with hypovitaminosis C (defined as < 23 μmol/L), and approximately 40% meeting criteria for vitamin C deficiency (defined as < 11 μmol/L) (9). Lower vitamin C levels are closely correlated with the severity of organ dysfunction in sepsis patients (10). The study by Marik et al. (11) proposed the “sepsis cocktail therapy” including high-dose vitamin C combined with hydrocortisone and thiamine demonstrated that this combination regimen significantly reduced mortality in sepsis patients. This research sparked extensive worldwide exploration into the potential therapeutic value of vitamin C in sepsis or septic shock.

However, current clinical studies on vitamin C application in sepsis patients present complex and contradictory outcomes. While some studies demonstrated positive outcomes, a systematic review encompassing 5,957 patients indicated that supplementation with vitamin C and magnesium could reduce short-term mortality in sepsis patients (12). However, negative findings have also been reported. The LOVIT trial revealed that high-dose vitamin C administration was associated with increased incidence of persistent organ dysfunction or even mortality compared with placebo (13). More recently, the C-EASIE trial randomized 292 emergency department patients with sepsis to intravenous vitamin C or placebo, finding no significant improvement in sequential organ failure assessment (SOFA) scores (14). Such heterogeneity has resulted in considerable controversy regarding the role of vitamin C in sepsis management. The Surviving Sepsis Campaign issued a weak recommendation against the routine use of intravenous vitamin C in adults with sepsis or septic shock (15). Meanwhile, significant variations exist in the dosage and administration regimens of vitamin C, which may be one of the important reasons for the inconsistent results. Incorporating the latest clinical research, the investigators conducted an exploratory meta-analysis to re-evaluate the application of vitamin C in sepsis or septic shock patients. With 28-day mortality rate, SOFA scores from baseline to 72 h or 96 h (ΔSOFA), urine output in the 24-h, hospital length of stay, ICU length of stay, the dosage and duration of vasopressors, and occurrence of adverse events as outcomes, this study compared the safety and therapeutic efficacy of different dosages of intravenous vitamin C as adjunctive therapy for patients with sepsis or septic shock.

## Materials and methods

### Study protocol and registration

This meta-analysis was conducted following the Preferred Reporting Items for Systematic Reviews and Meta-Analyses (PRISMA) guidelines (16). The protocol was prospectively registered on PROSPERO (CRD420261367808).

### Data sources and search strategy

Literature published from database inception to June 1, 2025, was systematically searched across PubMed, Embase, Scopus, and the Cochrane Library. To ensure comprehensive literature retrieval, references cited in the original studies were manually screened. Unpublished clinical trials were concurrently identified through ClinicalTrials.gov. The search strategy was as follows: [(sepsis OR “septic shock” OR “systemic inflammatory response syndrome” OR SIRS OR “sepsis syndrome”) AND (“vitamin C” OR “ascorbic acid” OR ascorbate) AND (“treatment” OR “therapy” OR “therapeutic” OR “management”)] AND (“randomized controlled trial” OR “controlled clinical trial” OR “randomized” OR “placebo” OR “clinical trials as topic” OR “randomly” OR “trial”).

### Inclusion criteria

The inclusion criteria were as follows: (1) Study type: Randomized controlled trials (RCTs); (2) Participants: Sepsis patients aged ≥ 18 years old with or without shock; (3) Interventions: compared the intravenous use of vitamin C at any dosage against standard sepsis care with or without placebo. For three-arm trials that included a vitamin C monotherapy arm, a combination therapy arm, and a placebo arm, only the vitamin C monotherapy and placebo arms were extracted for analysis; (4) Outcome measures: 28-day mortality rate, change in SOFA score from baseline to the earliest reported time point between 72 and 96 h (ΔSOFA), urine output in the 24-h, hospital length of stay, ICU length of stay, the dosage and duration of vasopressors, the level of vitamin C and adverse events. Exclusion criteria were as follows: (1) Study subjects were pediatric sepsis patients under 18 years old or pregnant women; (2) Review articles; (3) Case reports; (4) Animal studies; (5) Conference abstracts; (6) Studies with inaccessible full texts; (7) Duplicate publications using identical research data; (8) Administration of vitamins via oral route or nasogastric tube; (9) Studies in which vitamin C was administered in combination with other therapies (e.g., thiamine, corticosteroids, or other antioxidants), except for three-arm trials with a separate vitamin C monotherapy arm as specified in the Inclusion criteria, from which only the monotherapy and placebo arms were extracted.

### Study selection

Two investigators independently identified and screened relevant literature according to the predefined inclusion and exclusion criteria. Any disagreements arising during the screening process were resolved through careful deliberation with a third investigator.

### Data extraction

Two investigators independently extracted: first author, publication year, study design, sample size, baseline characteristics, vitamin C dosage, 28-day mortality (or in-hospital mortality as surrogate), vasopressor duration and dosage (norepinephrine equivalents), ICU and hospital length of stay (days), SOFA score (baseline to 72–96 h), 24-h urine output, plasma vitamin C concentration, and adverse events. Median (IQR) data were converted to mean ± SD using validated formulas (17). For three-arm trials, only monotherapy and placebo arms were extracted. Disagreements were resolved by a third investigator; unavailable data were recorded as NA.

### Risk of bias

The methodological quality of included RCTs was assessed independently by two reviewers using the Cochrane Risk of Bias 2.0 (RoB 2) tool, which evaluates bias across five domains: randomization process, deviations from intended interventions, missing outcome data, measurement of the outcome, and selection of the reported result.

### Clinical outcomes

The primary outcome was 28-day mortality. Collected 28-day mortality reported in the articles; if unavailable, report in-hospital mortality. When the mortality timeframe was unspecified, in-hospital mortality was presumed. Secondary outcomes included plasma concentration of vitamin C, urine output in the 24-h, hospital length of stay, ICU length of stay, the dose and duration of vasopressor, and changes in SOFA score. Serious adverse events related to intravenous vitamin C administration were simultaneously analyzed.

### Subgroup analysis

The dosage of vitamin C and the conditions of patients are major causes of outcome inconsistency. Exploratory subgroup analysis was conducted to identify potential sources of heterogeneity and generate hypotheses for future research. Based on key clinical trial findings (18–21), we compared the following interventions: very high dose vitamin C (≥ 12 g/day, or ≥ 150 mg/kg/day); high dose vitamin C (< 12 g, ≥ 6 g/day, or < 150 mg/kg/day, ≥ 75 mg/kg/day); low dose vitamin C (< 6 g/day, or < 75 mg/kg/day). The severity of patient illness is a major determinant of treatment response and a potential source of heterogeneity. Since no universally accepted SOFA threshold exists for distinguishing “moderate” from “severe” sepsis in the context of treatment effect modification, we adopted a data-driven approach. The median baseline SOFA score across all included studies was 9.75. Included studies with a mean baseline SOFA score ≥ 10 were classified into the higher-severity subgroup (*n* = 6 studies, analogous to “severe sepsis”), while those with mean baseline SOFA < 10 were classified into the lower-severity subgroup (*n* = 7 studies, analogous to “general sepsis”). The duration of vitamin C administration also varied across studies. Based on the administration regimens reported in the included studies, we categorized patients into a ≤ 3-day group and a > 3-day group for exploratory subgroup analysis. According to the number of participating institutions, we compared the single-center group with multi-center group (participating hospitals ≥ 2).

#### Quality of evidence

The quality of evidence for the outcomes was determined using the grading of recommendations assessment, development and evaluation (GRADE) approach (22). To achieve transparency and clarity, the GRADE system classifies the certainty of evidence in one of four grades: High: Further research is very unlikely to change our confidence in the estimate of effect. Moderate: Further research is likely to have an important impact on our confidence in the estimate of effect and may change the estimate. Low: Further research is very likely to have an important impact on our confidence in the estimate of effect and is likely to change the estimate. Very low: Any estimate of effect is very uncertain. Finally, the GRADE results summary table generated by GRADEpro GDT presents a summary of the study findings along with the corresponding GRADE ratings (23).

### Statistical analysis

Data analysis was performed using Review Manager (Version 5.4). Dichotomous data were analyzed using relative risk (RR) with 95% confidence interval (CI), while continuous data were analyzed using mean difference (MD) or standardized mean difference (SMD). Heterogeneity among study results was assessed using the *I*^2^. When *p* ≥ 0.1 and *I*^2^ < 50%, a fixed-effects model was employed; When *P* < 0.1 and *I*^2^ ≥ 50%, a random-effects model was applied, and sensitivity analysis was conducted to identify sources of heterogeneity. A two-sided *P* < 0.05 was considered statistically significant between groups.

## Results

### Literature search results and characteristics

**FIGURE 1:**
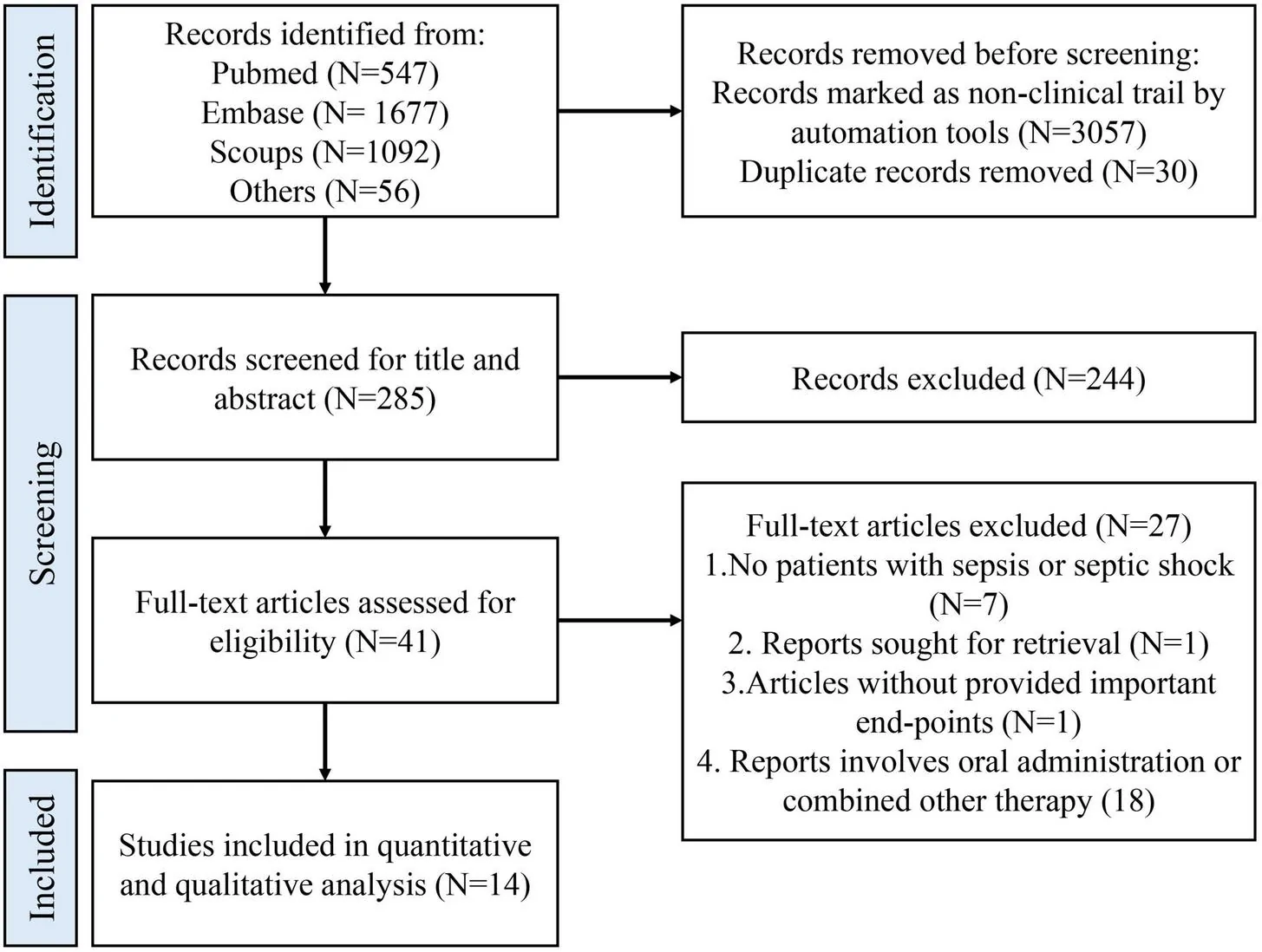
The selection flow diagram.

As shown in Figure 1, about 3,372 relevant articles were initially identified. After removing duplicates and non-clinical trial articles, 285 articles remained. Following title and abstract screening, 41 articles were retained. After full-text assessment, the following exclusions were applied: (1) studies without sepsis patients (*N* = 7), (2) retracted articles (*N* = 1), (3) studies with unavailable critical endpoint data (*N* = 1), (4) studies involving combination therapies or oral administration routes (*N* = 18). Ultimately, 14 randomized controlled trials were included for meta-analysis (13, 14, 20, 21, 24–33).

The characteristics of included studies are presented in Table 1. All 14 included studies were randomized controlled trials, encompassing a total of 1,958 patients. Among them, 991 patients were allocated to the vitamin C group and 968 to the Placebo group [One additional patient underwent randomization under deferred consent and died before consent was obtained. The research ethics board allowed the inclusion of this patient only in the primary analysis (13)]. The sample sizes of the included studies ranged from 24 to 862 patients. Overall, the mean age ranged between 52 and 71 years, and 764 (39.02%) participants were female. The mean SOFA score at baseline among patients was 8.76. The dose and duration of vitamin C administration differed across the included studies, as detailed in Table 1 and Supplementary Table 1. The definitions of sepsis, severe sepsis, and septic shock were independently established in each included study.

**TABLE 1:** Characteristics of included studies.

References	Group	Age, year Mean (SD)	Gender, female (*N*/total)	SOFA score Mean (SD)	APACHE II score Mean (SD)	Vitamin C level (umol/L)	Urine (ml) Mean (SD)	Vitamin C dosage
Vandervelden et al. (14)	Vitamin C group	64.7 (16.2)	50/147	4.9 (3.5)	NA	NA	NA	6 g/24 h, q6h, for 4 days
	Control group	67.0 (13.9)	53/145	5 (3.2)	NA	NA	NA	–
Li et al. (24)	High-dose vitamin C group	72.0 (14.7)	6/20	8.5 (3.5)	NA	17.3 (2.13)	2,020 (750.81)	150 mg/kg/24 h, q6h, for 4 days
	Low-dose vitamin C group	63.3 (17.1)	8/20	9 (4.74)	NA	18.61 (3.1)	2,242 (272.51)	50 mg/kg/24 h, q6h, for 4 days
	Control group	67.8 (13.5)	2/18	10.22 (3.42)	NA	23.48 (11.46)	1,748.33 (542.39)	–
Jiang et al. (25)	Vitamin C group	53.3 (13.2)	20/42	NA	NA	NA	NA	3 g/24 h, once daily for 3 days
	Control group	53.3 (13.2)	19/22	NA	NA	NA	NA	–
Yanase et al. (26)	Vitamin C group	64.0 (17.1)	3/15	7 (6, 8)*	59 (28.89)	19.0 (5.19)	60 (46.67) [ml/h]	60 g/24 h, BID, not report time
	Control group	70.0 (17.0)	6/15	7 (5, 8)*	57 (5.92)	30.0 (7.41)	100 (64.44) [ml/h]	–
Wacker et al. (27)	Vitamin C group	68.9 (14.7)	30/60	10 (7, 11)*	22 (8.52)	NA	NA	1 g/24 h, for 3 days
	Control group	73.0 (14.2)	31/64	9 (7, 12)*	23 (8.52)	NA	NA	–
El Driny et al. (29)	Vitamin C group	53.0 (23.3)	11/20	12.1 (1.6)	23.2 (4.7)	21.4 (10.6)	NA	6 g/24 h, q6h, for 4 days
	Control group	52.1 (18.8)	8/20	12.7 (2.1)	22.6 (5.4)	22.5 (12.3)	NA	–
Rosengrave et al. (28)	Vitamin C group	69.0 (8.9)	4/20	8.5 (6.8, 11)*	85 (16.3)	10.0 (6.67)	NA	100 mg/kg/24 h, for 3 days (median: 8 g/d)
	Control group	66.0 (11.1)	9/20	9 (7.8, 10)*	81 (18.52)	8.2 (4.67)	NA	–
Lamontagne et al. (13)	Vitamin C group	65.0 (14.0)	151/429	10.2 (3.4)	24.2 (7.4)	20.6 (70.6)	767 (820)	200 mg/kg/24 h, for 3 days, q6h
	Control group	65.2 (13.8)	173/433	10.1 (3.7)	24.1 (7.9)	19.1 (39.7)	751 (799)	–
Labbani-Motlagh et al. (32)	Vitamin C group	57.84 (14.722)	15/37	2.89 (1.329)	NA	NA	NA	12 g/24 h, for 4 days
	Control group	58.89 (14.460)	17/37	3.05 (1.373)	NA	NA	NA	–
Mahmoodpoor et al. (33)	Vitamin C group	56.93 (12.3)	18/40	12.54 (2.65)	24.5 (5.35)	20.63 (12.74)	NA	60 mg/kg/day, for 3 days
	Control group	58.25 (13.1)	16/40	10.7 (2.70)	22.7 (4.26)	22.77 (13.56)	NA	–
Zhang et al. (30)	Vitamin C group	66.3 (11.2)	12/27	3.5 (3, 6.8)*	14 (3.7)	NA	NA	24 g/24 h, daily for 7 days
	Control group	67.0 (14.3)	7/29	2 (3, 5)*	13 (4.07)	NA	NA	–
Fowler et al. (20)	Vitamin C group	54.0 (20.7)	39/84	9.8 (3.2)	NA	22.0 (22.96)	14.1 (14.5) [ml/kg/h]	200 mg/kg/24 h, for 3 days
	Control group	57.0 (19.3)	38/83	10.3 (3.1)	NA	22.0 (26.67)	10.5 (11.7) [ml/kg/h]	–
Zabet et al. (31)	Vitamin C group	64.1 (16.0)	4/14	11.78 (2.22)	19.07 (5.18)	NA	3,329.64 (1,746.3)	100 mg/kg/24 h, for 3 days, q6h
	Control group	63.7 (13.8)	3/14	12.35 (3)	23.0 (5.61)	NA	3,357.14 (2,022.93)	
Fowler et al. (21)	High-dose vitamin C group	49–92^&^	4/8	10.8 (4.4)^#^	24.0 (5.25)	17.0 (28.89)	NA	200 mg/kg/24 h, for 3 days
	Low-dose vitamin C group	30–70^&^	3/8	10.1 (2)^#^	20.4 (5.25)	16.7 (10.37)	NA	50 mg/kg/24 h, for 3 days
	Control group	54–68^&^	4/8	13.3 (2.9)^#^	20.4 (3.5)	20.2 (25.19)	NA	–

^&^Range,

*Median (IQR); ^#^MD (SE). SOFA, sequential organ failure assessment.

### Risk of bias assessment for included studies

The methodological quality of the 14 included RCTs was assessed independently by two reviewers using the Cochrane Risk of Bias 2.0 (RoB 2) tool, which evaluates bias across five domains: the randomization process, deviations from intended interventions, missing outcome data, measurement of the outcome, and selection of the reported result. The overall risk of bias was low across most domains (Supplementary Figure 1).

### Meta-analysis of primary outcomes

All 14 included trials reported either 28-day mortality rates or death counts (13, 14, 20, 21, 24–33). One study (14) reported the estimated 28-day mortality rate, from which the number of deaths was inferred. Two studies (21, 24) included separate high-dose and low-dose vitamin C arms versus a common placebo group; for each study, we pooled the mortality data from both vitamin C arms into a single estimate before inclusion in the primary meta-analysis.

**FIGURE 2:**
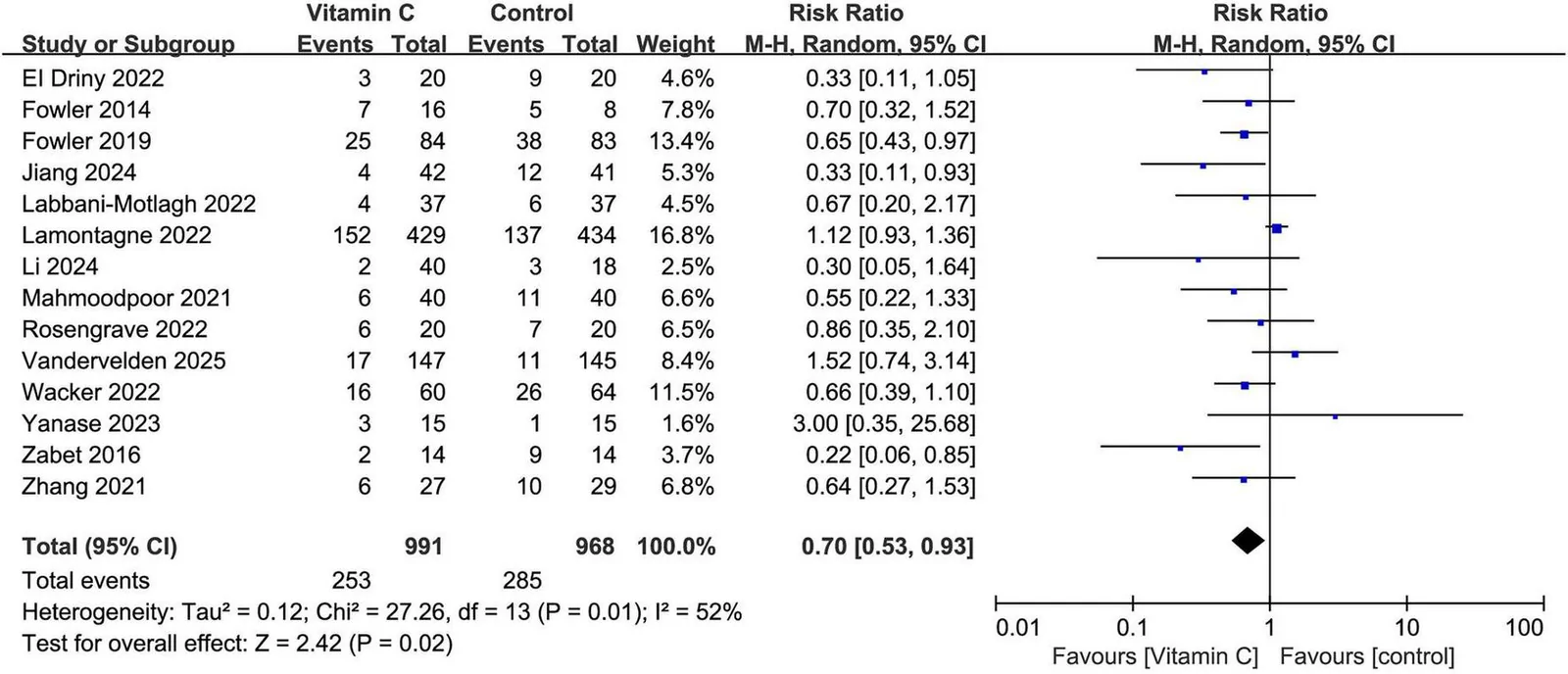
Forest plot displaying the effect of vitamin C treatment group on the mortality.

Pooled data analysis revealed a mortality rate of 29.44% (285/968) in the control group versus 25.53% (253/991) in the vitamin C group. Compared with the control group, intravenous vitamin C administration was associated with reduced overall mortality (RR 0.70; 95% CI: 0.53 to 0.93; *P* = 0.02; *I*^2^ = 52%) (Figure 2). The funnel plot showed slight asymmetry, with more studies on the left side, although Egger’s test was not significant (*P* = 0.07, Supplementary Figure 2).

### Subgroup analysis

#### Dose and duration of vitamin C

Seven studies (13, 20, 21, 24, 26, 30, 32) employed very high-dose intravenous vitamin C administration, four studies (14, 28, 29, 31) utilized high-dose intravenous vitamin C administration, and another five studies (21, 24, 25, 27, 33) adopted low-dose intravenous vitamin C administration. Data from the very high-dose and low-dose intravenous vitamin C groups in two studies were analyzed separately within their respective subgroups (21, 24).

Low-dose intravenous vitamin C administration was significantly associated with reduced overall mortality (RR 0.57; 95% CI 0.39 to 0.83; *P* = 0.003; *I*^2^ = 0%). In contrast, the very high dose group (RR 0.83; 95% CI 0.59 to 1.18; *P* = 0.31; *I*^2^ = 42%) and the high-dose group (RR 0.63; 95% CI 0.27 to 1.48; *P* = 0.29; *I*^2^ = 66%) did not significantly reduce mortality. Subgroup difference testing among the three groups revealed a consistent trend (*P* = 0.34, *I*^2^ = 7.9%) (Supplementary Figure 3). The duration of vitamin C administration also varied across studies. Based on the administration regimens reported in the included studies, patients were categorized into a ≤ 3-day group and a > 3-day group. Subgroup analysis indicated that intravenous vitamin C administration for ≤ 3 days reduced mortality in patients with sepsis (RR 0.67; 95% CI 0.48 to 0.95; *P* = 0.03; *I*^2^ = 63%). However, the high heterogeneity within the subgroup warrants cautious interpretation of these results (Supplementary Figure 4).

#### SOFA score

Of the 14 included studies, 13 reported overall baseline SOFA scores and were included in this subgroup analysis. Seven studies (14, 24, 26–28, 30, 32) with baseline SOFA scores < 10 were included in the general sepsis group, six studies (13, 20, 21, 29, 31, 33) with baseline SOFA scores ≥ 10 were assigned to the severe sepsis subgroup. One study (25) did not report an overall SOFA score and was excluded. In the severe sepsis subgroup, intravenous vitamin C showed a potential mortality benefit that approached statistical significance (RR 0.64; 95% CI 0.41 to 1.01; *P* = 0.05; *I*^2^ = 69%), whereas no significant benefit was observed in the general sepsis subgroup (RR 0.82; 95% CI 0.57 to 1.17; *P* = 0.34; *I*^2^ = 11%). The subgroup difference was not significant (*P* = 0.42; *I*^2^ = 0%) (Supplementary Figure 5).

#### The number of participating institutions

To further analyze the impact of the number of participating hospitals on the mortality of sepsis patients receiving adjunctive vitamin C therapy, a subgroup analysis was conducted comparing single-center and multi-center studies. Among the studies included, nine studies were single-center, and five studies involved multi-center. In the single-center subgroup, intravenous vitamin C administration was observed to significantly reduce 28-day mortality in sepsis patients (RR 0.54; 95% CI 0.38 to 0.78; *P* = 0.0009; *I*^2^ = 0%). In contrast, no similar effect was observed in the multi-center subgroup (RR 0.87; 95% CI 0.62 to 1.22; *P* = 0.43; *I*^2^ = 62%) (Supplementary Figure 6).

### Meta-analysis of secondary outcomes

#### The use of vasoactive drug

**FIGURE 3:**
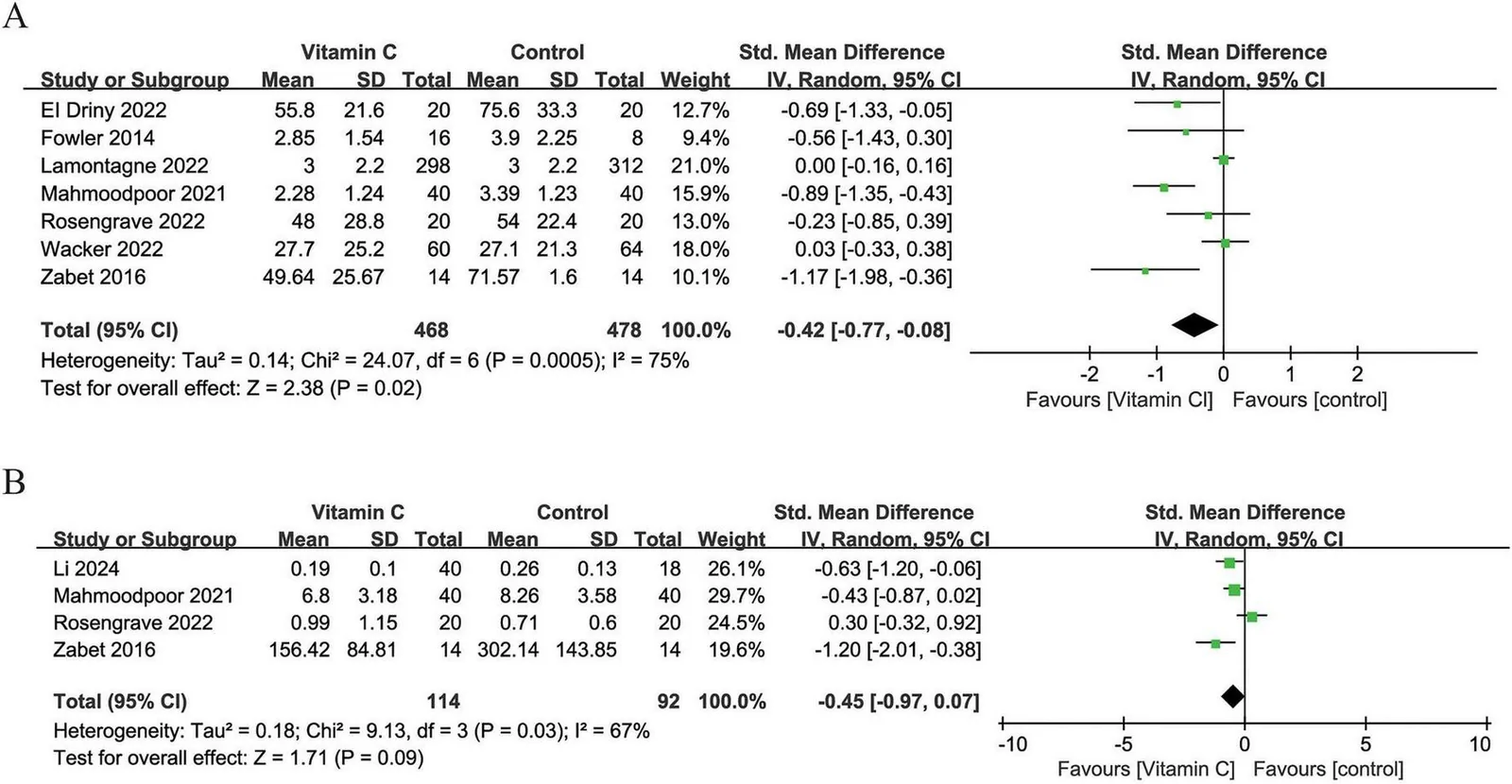
Forest plot displaying the effect of vitamin C on the application time and dose of vasoactive drugs. **(A)** Forest plot displaying the effect of vitamin C on the application time of vasoactive drugs. **(B)** Forest plot displaying the effect of vitamin C on the dose of vasoactive drugs.

Seven studies (13, 21, 27–29, 31, 33) involving 946 patients reported the duration of vasoactive drug use. Heterogeneity testing indicated significant heterogeneity (*P* < 0.05, *I*^2^ = 75%). A meta-analysis using a random-effects model showed significant difference in the duration of vasoactive drug use between the vitamin C group and the control group (SMD = −0.42, 95% CI −0.77 to −0.08, *P* = 0.02) (Figure 3A). However, the effect was limited by the high heterogeneity.

Four studies (24, 28, 31, 33) involving 206 patients reported the dose of vasoactive drugs used. Heterogeneity tests indicated significant heterogeneity (*P* < 0.05, *I*^2^ = 67%). The meta-analysis using a random-effects model showed no significant difference in the dose of vasoactive drugs between the vitamin C group and the control group (SMD = −0.45, 95% CI −0.97 to 0.07, *P* = 0.09) (Figure 3B). Sensitivity analysis after excluding the Rosengrave et al. (28) study reduced heterogeneity (*P* = 0.26, *I*^2^ = 25%) and suggested a potential dose reduction in the vitamin C group (SMD = −0.64, 95% CI −1.02 to −0.26, *P* = 0.001). However, given the *post-hoc* nature of this analysis, this finding should be interpreted with caution.

#### Changes in SOFA scores

**FIGURE 4:**
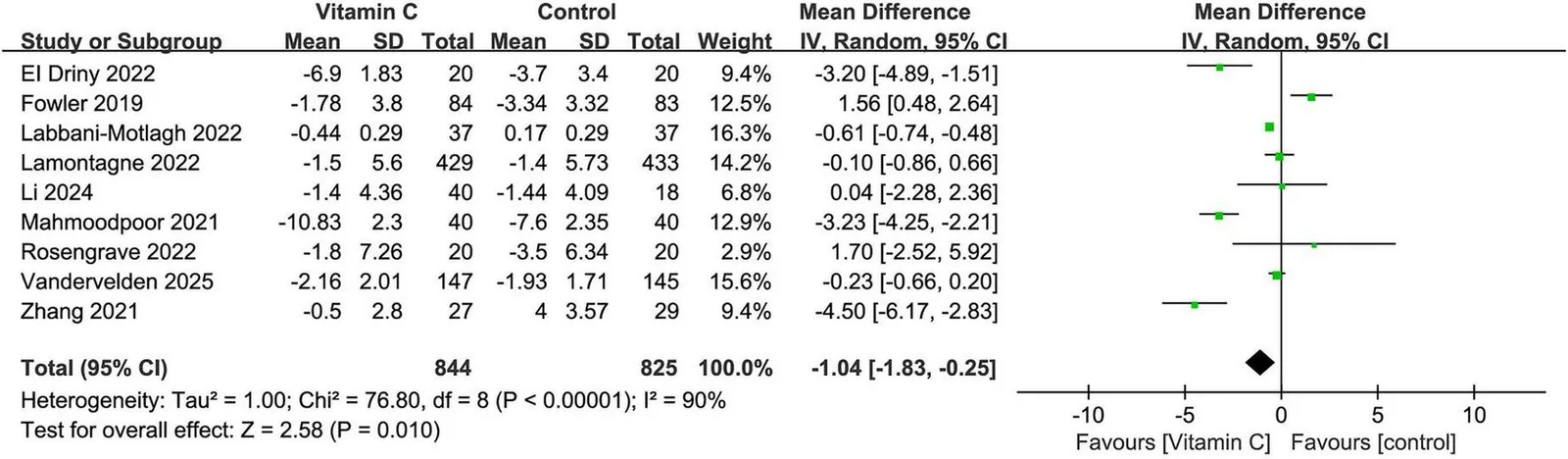
Forest plot displaying the effect of vitamin C on the change of sequential organ failure assessment (SOFA) scores from baseline to 72 h or 96 h post-treatment.

Nine studies (13, 14, 20, 24, 28–30, 32, 33) involving 1,669 patients reported SOFA scores before and after treatment. The difference in SOFA scores before and after treatment (ΔSOFA) was analyzed. The heterogeneity test revealed significant heterogeneity (*P* < 0.05, *I*^2^ = 90%). A meta-analysis using the random-effects model showed that the vitamin C group was associated with a significantly greater reduction in SOFA scores (MD = −1.04, 95% CI −1.83 to −0.25, *P* = 0.01) (Figure 4). The high heterogeneity, however, compromises the reliability of this result, which should be interpreted cautiously.

#### Vitamin C levels after treatment

**FIGURE 5:**
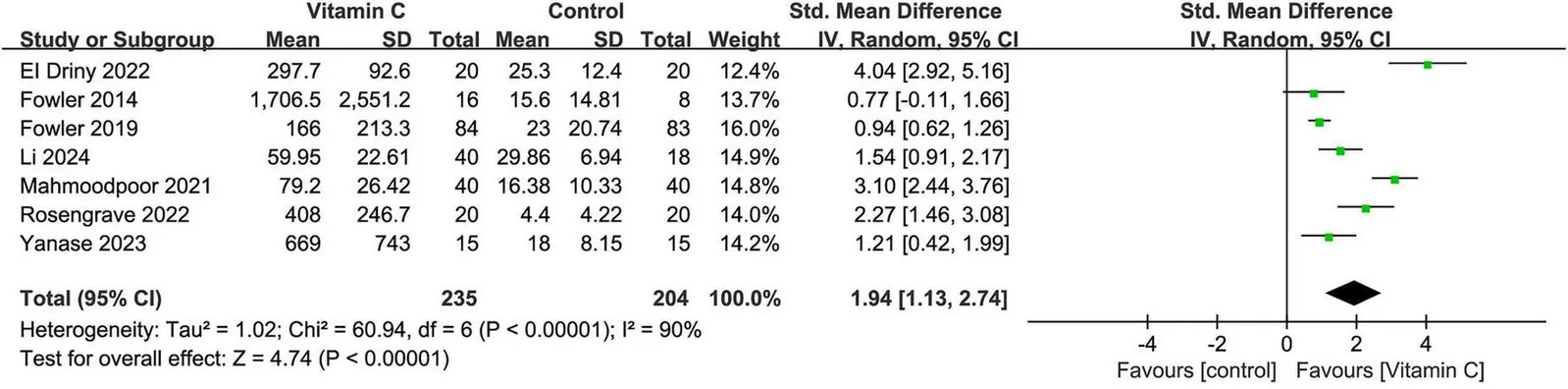
Forest plot displaying the effect of vitamin C on the change of vitamin C level from baseline to 72 h or 96 h post-treatment.

Seven studies (20, 21, 24, 26, 28, 29, 32) involving 439 patients reported vitamin C levels before and after treatment. Analysis of the difference in vitamin C levels after treatment revealed significant heterogeneity through heterogeneity testing (*P* < 0.05, *I*^2^ = 90%). A meta-analysis was performed using the random-effects model, demonstrating that intravenous vitamin C administration significantly increased serum vitamin C levels (SMD = 1.94, 95% CI 1.13 to 2.74, *P* < 0.001) (Figure 5 and Supplementary Figure 7). The high heterogeneity compromised the reliability of the results.

#### Hospital length of stay

Eight studies (13, 14, 24, 26–28, 30, 32) involving 1,536 patients analyzed hospital length of stay. Heterogeneity testing indicated no significant heterogeneity (*P* = 0.36, *I*^2^ = 9%). Meta-analysis using the fixed-effects model indicated that the vitamin C group did not reduce patients’ length of hospital stay (MD = 0.50, 95% CI −0.82 to 1.83, *P* = 0.45) (Supplementary Figure 8).

#### ICU length of stay

Ten studies (13, 14, 21, 24, 27–31, 33) involving 1,604 patients analyzed ICU length of stay. Heterogeneity test revealed no significant heterogeneity (*P* = 0.09, *I*^2^ = 40%). Meta-analysis using the fixed-effects model demonstrated that the vitamin C group did not reduce patients’ ICU length of stay (MD = −0.15, 95% CI −0.75 to 0.45, *P* = 0.62) (Supplementary Figure 9).

#### Post-treatment urine output

The five included randomized controlled trials (13, 20, 24, 26, 31), comprising 1,145 patients, reported pre-treatment and post-treatment urine output. One study reported the mean urine output during the treatment period (31). With no statistically significant difference in baseline urine output between the two groups, a meta-analysis was directly performed on the post-treatment urine output data. The heterogeneity test revealed significant heterogeneity (*P* < 0.05, *I*^2^ = 70%). Using a random-effects model for meta-analysis, no statistically significant difference was found in post-treatment urine output between the two groups (SMD = 0.29, 95% CI −0.04 to 0.63, *P* = 0.08) (Supplementary Figure 10). Re-analysis after excluding Li et al. (24) reduced heterogeneity (*P* = 0.31, *I*^2^ = 15%), but still showed no statistically significant difference (SMD = 0.05, 95% CI −0.07 to 0.17, *P* = 0.39).

#### Incidence of adverse events

During adjunctive therapy with intravenous vitamin C administration, attention should also focus on the incidence of adverse events. Comprehensive analysis revealed that twelve studies (13, 14, 20, 21, 24, 26–28, 30–33) reported adverse events, but only five documented (13, 26, 27, 32, 33) treatment-related adverse events. These included hypernatremia, moderate hemolysis, gastrointestinal bleeding, non-ST-elevation myocardial infarction, hypoglycemia, allergic reactions, diabetic ketoacidosis, soft tissue swelling, and pain (Supplementary Table 2). These adverse events could be managed with symptomatic treatment and did not cause any patients to discontinue therapy. The remaining seven research reports observed no adverse events associated with vitamin C administration during their study periods.

#### Grading the evidence

The summary of findings and the associated GRADE quality of evidence ratings for this review are presented in Supplementary Table 3.

## Discussion

Vitamin C directly scavenges oxygen free radicals (ROS) (10, 34), promotes catecholamine and vasopressin synthesis (8, 35, 36), enhances immune cell function (37, 38), improves microcirculation (7), and suppresses inflammatory pathways such as NF-κB while reducing pro-inflammatory cytokine levels (39). However, it has been reported that the plasma concentration of vitamin C mostly tends to decline in sepsis patients (9). These mechanisms constitute the theoretical foundation for vitamin C application in sepsis treatment. Our findings indicated that intravenous vitamin C administration, regardless of dose, effectively elevates plasma vitamin C levels with a favorable safety profile and no significant occurrence of adverse events. Exogenous supplementation of vitamin C can promote the synthesis of vasopressors (8, 35) and enhance the body’s responsiveness to norepinephrine and angiotensin II (40, 41). Meanwhile, it improves organ dysfunction in patients with sepsis by protecting mitochondria (42, 43). These findings are consistent with some results of this study, which demonstrated that intravenous administration of vitamin C can shorten the duration of vasopressor use. The observed reduction in SOFA scores, however, should be interpreted with caution given the substantial heterogeneity (*I*^2^ = 90%) across the included studies.

The primary outcome of this study showed that intravenous vitamin C was associated with lower mortality for sepsis patients, which should be interpreted with extreme caution. First, the benefit was not robust across high-quality studies. The two largest, double-blind, multicenter RCTs (LOVIT and C-EASIE) did not show a mortality benefit and, in fact, numerically suggested potential harm (13, 14). Our subgroup analysis of multicenter trials, which are generally less prone to bias, showed no significant mortality reduction. Second, the observed benefit is primarily driven by smaller, single-center studies. This discrepancy may be attributed to factors such as a higher likelihood of publication bias in single-center studies (44), their generally smaller sample sizes (45), and potentially lower methodological quality relative to multicenter RCTs (46). Nevertheless, these outcome differences reflect variations in study methodology and patient populations, and do not necessarily negate the therapeutic potential of intravenous vitamin C. On the contrary, future large-scale, multicenter randomized controlled trials are warranted to validate the real-world efficacy of vitamin C.

The administration methods of vitamin C include intravenous administration and oral administration. Compared to enteral delivery, intravenous administration of vitamin C achieves higher plasma levels (47). Sepsis patients often experience concurrent gastrointestinal dysfunction. Coupled with intestinal transport saturation and impaired mucosal function leading to restricted intestinal absorption, enteral supplementation of vitamin C results in limited plasma concentrations (47). This study exclusively analyzed research related to intravenously administered vitamin C. The result indicated that intravenous vitamin C administration, regardless of dose, effectively elevates plasma vitamin C levels with a favorable safety profile and no significant occurrence of adverse events. However, the subgroup analyses showed that the benefit of vitamin C was only observed in the low-dose and shorter-duration (≤ 3 days) subgroups. This might be because vitamin C absorption and utilization in the body follow a saturation mechanism. Once a certain plasma concentration is reached, increasing the dose further does not proportionally increase intracellular vitamin C concentrations but instead results in renal excretion of the excess (47, 48). More importantly, at supra-physiological concentrations, ascorbate can act as a reducing agent for metal ions such as iron, potentially catalyzing the Fenton reaction and generating hydroxyl radicals, which may exacerbate rather than attenuate oxidative injury (49, 50). This pro-oxidant switch could theoretically offset the antioxidant benefits of vitamin C at very high doses, potentially explaining the lack of efficacy observed in the high-dose and very high-dose subgroups. Additionally, mitochondrial dysfunction is a key driver of organ failure in sepsis (51). Given that excessive oxidative stress can exacerbate mitochondrial damage, high-dose vitamin C—through its pro-oxidant effects—could theoretically worsen, rather than improve, mitochondrial function (52, 53). This further supports a “U-shaped” or “threshold” dose-response relationship, where only moderate doses confer benefit, while excessive doses may be detrimental. These mechanistic considerations, while speculative, underscore the need for carefully designed dose-finding studies to establish the optimal vitamin C regimen in sepsis, as well as the importance of measuring baseline vitamin C levels to identify patients who are truly deficient and most likely to benefit from replacement therapy.

The severity of patients enrolled in RCTs significantly influences study outcomes. This analysis included patients with sepsis and/or septic shock, though some studies failed to distinguish between cohorts of sepsis patients and septic shock patients. The SOFA score serves as a crucial tool for assessing organ function and disease severity in sepsis patients. Studies indicate that an increase of ≥ 2 points in SOFA score from baseline correlates with elevated mortality risk, approximately ≥ 10% (54). To further analyze therapeutic efficacy of vitamin C across sepsis subgroups, patients were stratified into severe sepsis (SOFA ≥ 10) and general sepsis (SOFA < 10) groups based on mean baseline SOFA scores. Subgroup analysis revealed that in the severe sepsis subgroup (SOFA ≥ 10), intravenous vitamin C was associated with a potential mortality benefit that approached statistical significance, whereas no such benefit was observed in the general sepsis subgroup. This result, however, aligns with the biologically plausible hypothesis that patients with more severe sepsis may have greater depletion of endogenous antioxidant reserves and thus may derive more substantial benefit from exogenous vitamin C supplementation. Severe sepsis patients frequently present with profound vitamin C deficiency due to increased metabolic consumption, reduced intake, and increased urinary losses (9), creating a state where exogenous supplementation may function as replacement therapy in deficiency states rather than as a pharmacological agent in non-deficient individuals. Furthermore, vitamin C plays a critical role in restoring vascular responsiveness to vasopressors (8, 35) and preserving microcirculatory function (7), mechanisms particularly relevant in more hemodynamically unstable patients with higher SOFA scores. Nevertheless, the high heterogeneity (*I*^2^ = 69%) in the severe sepsis subgroup warrants cautious interpretation.

The results of this study suggested it has shorter duration of vasopressor use in the vitamin C group than control group. This is similar to previous studies that have shown vitamin C administration significantly shortens the duration of vasopressor use (55, 56). Some studies have reported that vitamin C can restore vascular responsiveness to vasoactive agents; theoretically, the duration and dose of vasopressor use in the vitamin C group should be lower than those in the control group. However, few studies reported data on vasopressor dose. This meta-analysis indicated that vitamin C use did not significantly reduce the dose of vasopressor administration. The output of urine is an important clinical indicator of renal function. Many sepsis patients experience renal impairment, and 24-h urine output can reflect the recovery of renal function in these patients. Five RCTs reporting post-treatment urine output were included in this analysis. The results showed no statistically significant difference between the vitamin C group and the control group. Furthermore, this study found that the use of vitamin C did not significantly reduce the length of hospital stay or ICU stay, which is consistent with previous researches (57, 58). This may be because factors such as healthcare systems, complications, and even patient preferences rather than a single medication have a greater influence on hospitalization duration. In summary, while there is a strong theoretical basis for vitamin C in improving outcomes in sepsis patients, clinical trial results have been contradictory. This suggests that vitamin C may reduce mortality through mechanisms such as enhancing immune function and promoting tissue healing, effects which may not be directly reflected in macroscopic organ function. We anticipate that future large-scale clinical studies and basic experiments will further analyze and validate the efficacy of vitamin C in sepsis patients and elucidate its potential underlying mechanisms.

Adverse reactions also constitute a significant concern in the clinical application of vitamin C. A meta-analysis indicated higher adverse event incidence in the vitamin C group (55). Careful differentiation is required between vitamin C-related adverse events and sepsis-related adverse events during assessment. vitamin C-associated adverse events identified in this study included hypernatremia, hemolysis, and allergic reactions. These events improved with symptomatic treatment and did not lead to patient withdrawal from the study. Meanwhile, the majority of studies reported no adverse reactions related to vitamin C administration during the research period.

There are several limitations in this meta-analysis. First, although the pooled analysis showed a mortality benefit associated with vitamin C, this finding was primarily driven by smaller single-center studies and was not confirmed in the two largest double-blind, multicenter RCTs (13, 14), which individually reported neutral or potentially harmful effects. This discrepancy warrants caution in interpreting the overall estimate. Second, the overall certainty of evidence for the primary outcome was rated as low according to the GRADE assessment, indicating that future well-designed studies are likely to have an important impact on our confidence in the estimate and may change the conclusion. Third, multiple subgroup analyses reduced the sample size within each stratum, limiting the precision and generalizability of these exploratory findings. Fourth, the inclusion of studies with small sample sizes may have inflated the treatment effect and influenced the overall results. Fifth, substantial heterogeneity was observed for ΔSOFA and post-treatment vitamin C levels, likely reflecting between-study differences in baseline severity, timing of intervention, dosing regimens, and measurement protocols. These secondary outcomes should therefore be interpreted with caution and considered exploratory. Finally, the SOFA threshold of 10 used for subgroup analysis was data-driven, based on the median distribution of baseline SOFA scores across included studies, rather than derived from external validation; this threshold has not been prospectively validated as a treatment effect modifier. Future large-scale, multicenter RCTs with prespecified subgroup analyses are needed to confirm these hypothesis-generating findings.

## Conclusion

This systematic review and meta-analysis indicated that adjunctive vitamin C therapy may be associated with reduced mortality in sepsis or septic shock patients, elevates plasma vitamin C concentration, and consequently improves patient prognosis. No significant safety concerns were identified with intravenous vitamin C administration, as reported adverse events were manageable and did not lead to treatment discontinuation. However, the lack of benefit in large, high-quality multicenter RCTs, the suggestive evidence of publication bias, and the inconsistency across subgroups preclude a definitive conclusion of efficacy. Our findings should be considered hypothesis-generating, providing a rationale for future, rigorously designed, multicenter RCTs.

## Data Availability

The original contributions presented in this study are included in this article/Supplementary material, further inquiries can be directed to the corresponding author.
