# Supplementary material for: Intravenous vitamin C as micronutrient therapy in patients with sepsis or septic shock: an updated systematic review and meta-analysis of randomized controlled trials
Source: Front Nutr. 2026 Sep 4;13:1905726. doi: 10.3389/fnut.2026.1905726 (PMC13585581; doi:10.3389/fnut.2026.1905726)
Supplement: Supplementary file 1 [file Table_1.docx]

Supplementary table S1. Baseline characteristics of included studies

| Study  Author year | Patients | Severity score, mean | Country | Source of infection | Comparison | Other |
| --- | --- | --- | --- | --- | --- | --- |
| Vandervelden et al. (2025) | Sepsis or septic shock | SOFA score, 4.9 | Belgium  (eight center) | Respiratory, gastro-intestinal, urinary, skin or soft tissue, catheter, other | Vit C vs placebo | Protocol: NCT04747795 |
| Li et al. (2024) | Septic shock | SOFA score, 9.2 | China | Lungs, bloodstream, biliary tract, abdomen | High dose Vit C vs low dose Vit C vs placebo | - |
| Jiang et al (2024) | Sepsis | NA | China | NA | Vit C vs placebo | - |
| Yanase et al (2023) | Septic shock | SOFA score, 7 | Australia | Gastro-intestinal/biliary, primary bacteraemia, respiratory, soft tissue, urinary, other | Vit C vs placebo | Protocol: ACTRN12620000651987 |
| Wacker et al. (2022) | Septic shock | SOFA score, 9.5 | America | Pulmonary, urinary, gastrointestinal/biliary, soft tissue/skin, primary bacteremia, endocarditis, bacterial meningitis, other/unknown | Vit C vs placebo | Protocol: NCT03338569 |
| El Driny et al. (2022) | Sepsis | SOFA score, 12.4 | Egypt | Central venous catheter, urinary tract infection, abdominal, skin and soft tissue, infected diabetic foot | Vit C vs placebo | Protocol: NCT04029675 |
| Rosengrave et al. (2022) | Septic shock | SOFA score, 8.8 | New Zealand | Abdominal | Vit C vs placebo | Protocol: ACTRN12617001184369 |
| Lamontagne et al. (2022) | Sepsis | SOFA score, 10.1 | Canada,  France, New Zealand | Pulmonary, gastrointestinal or intra-abdominal, blood, skin or soft tissue, urinary, central nervous system, other | Vit C vs placebo | Protocol: NCT03680274 |
| Labbani-Motlagh et al. (2022) | COVID‑19  infection | SOFA score, 2.97 | Iran | Pneumonia | Vit C vs placebo | Protocol: IRCT20190917044805N2 |
| Mahmoodpoor (2021) | severe pneumonia | SOFA score, 11.58 | Iran | Pneumonia | Vit C vs placebo | Protocol: IRCT20190312043030N1 |
| Zhang et al. (2021) | Ill COVID-19 patients | SOFA score, 2.7 | China | Pneumonia | High-dose Vit C vs placebo | Protocol: NCT04264533 |
| Fowler et al. (2019) | Sepsis | SOFA score, 10 | United States | Thorax, abdomen, urinary tract, central nervous system, central venous catheter, unknown/other | Vit C vs placebo | Protocol: NCT02106975 |
| Zabet et al. (2016) | Septic shock | SOFA score, 12.1 | Iran | Abdominal, pulmonary, urinary tract, central line catheter, skin and soft tissue | Vit C vs placebo | - |
| Fowler et al. (2014) | Severe sepsis | SOFA score, 11.4 | United States | Pneumonia, spontaneous bacterial peritonitis, urinary tract infection, catheter sepsis, infected diabetic foot ulcer, skin, other | High dose Vit C vs low dose Vit C vs placebo | Protocol: NCT01434121 |

Notes: SOFA: Sequential organ failure assessment; vs: verse; Vit C: vitamin C; SD: standard deviation.

Supplementary table S2. Adverse events

| Study  Author year | Status | Vitamin C group | | Control group | |
| --- | --- | --- | --- | --- | --- |
|  |  | Events | Total | Events | Total |
| Vandervelden et al. (2025) | No events related with study | | | | |
| Li et al. (2024) | No vitamin C-related adverse events were observed | | | | |
| Jiang et al (2024) | NA | | | | |
| Yanase et al (2023) | Hypernatremia | 1 | 15 | 0 | 15 |
|  | Moderate hemolysis | 1 |  | 0 |  |
| Wacker et al. (2022) | Gastrointestinal bleed | 1 | 20 | 0 | 20 |
| El Driny et al. (2022) | NA | | | | |
| Rosengrave et al. (2022) | No vitamin C-related adverse events were observed | | | | |
| Lamontagne et al. (2022) | Non-ST elevation myocardial infarction | 1 | 429 | 0 | 433 |
|  | Hypoglycemic episode | 1 |  | 0 |  |
|  | Anaphylaxis | 1 |  | 0 |  |
|  | Diabetic ketoacidosis | 1 |  | 0 |  |
|  | Soft tissue swelling and burning pain | 0 |  | 1 |  |
| Labbani-Motlagh et al. (2022) | Allergic reaction | 1 | 37 | 0 | 37 |
| Mahmoodpoor (2021) | Hypotension and tachycardia | 3 | 40 | 0 | 40 |
| Zhang et al. (2021) | No vitamin C-related adverse events were observed | | | | |
| Fowler et al. (2019) | No vitamin C-related adverse events were observed | | | | |
| Zabet et al. (2016) | No vitamin C-related adverse events were observed | | | | |
| Fowler et al. (2014) | No patients were withdrawn due to study-related adverse events | | | | |

Supplementary table S3. GRADE summary of findings table

| **Number of studies** | **Number of patients** | | **Effect** | | **Certainty of evidence**  **(GRADE)** |
| --- | --- | --- | --- | --- | --- |
|  | **Intravenous Vitamin C** | **Control** | **Relative (95% CI)** | **Absolute (95% CI)** |  |
| **Incidence of 28-day mortality** | | | | | |
| 14 | 253/991 (25.5%) | 285/968 (29.4%) | **RR 0.70** (0.53 to 0.93) | **88 fewer per 1,000**  (from 138 fewer to 21 fewer) | ⨁⨁◯◯ Low ^a, b^ |
| **The use duration of vasoactive drug** | | | | | |
| 7 | 468 | 478 | - | **SMD 0.42 SD fewer**  (0.77 fewer to 0.08 fewer) | ⨁⨁◯◯ Low ^b, c^ |
| **The use total dose of vasoactive drug** | | | | | |
| 4 | 114 | 92 | - | **SMD 0.45 SD fewer**  (0.97 fewer to 0.07 more) | ⨁◯◯◯ Very low ^b, d, e^ |
| **ΔSOFA** | | | | | |
| 9 | 844 | 825 | - | **MD 1.04 lower**  (1.83 lower to 0.25 lower) | ⨁⨁◯◯ Low ^f^ |
| **Vitamin C levels after treatment** | | | | | |
| 7 | 235 | 204 | - | **SMD 1.94 SD higher** (1.13 higher to 2.74 higher) | ⨁⨁◯◯ Low ^f^ |
| **Hospital length of stay** | | | | | |
| 8 | 775 | 761 | - | **MD 0.5 higher** (0.82 lower to 1.83 higher) | ⨁⨁◯◯ Low ^g^ |
| **ICU length of stay** | | | | | |
| 10 | 813 | 791 | - | **MD 0.15 lower** (0.75 lower to 0.45 higher) | ⨁⨁◯◯ Low ^h, i^ |
| **Post-treatment urine output** | | | | | |
| 5 | 582 | 563 | - | **SMD 0.29 SD higher** (0.04 lower to 0.63 higher) | ⨁◯◯◯ Very low ^b, d, j^ |

Note: CI: confidence interval; MD: mean difference; RR: risk ratio; SMD: Standardized mean difference. a. There are two high-quality, multicenter, large-sample, randomized controlled trials included in the analysis—Lamontagne 2022 and Vandervelden 2025—demonstrated that intravenous vitamin C administration did not reduce mortality in patients with sepsis, which showed that the inconsistent with the results of other trials. b. I² statistics indicate the possible presence of moderate heterogeneity. c. Four studies reported the duration of vasoactive drug use in hours as the statistical unit, while the other three studies reported it in days. The use of days as a unit provides a broader range but is less precise and accurate compared to hours. d. The inconsistency in the statistical units used across studies affects the accuracy of the results. e. A total of seven studies reported the total duration of vasoactive drug administration, whereas only four studies reported the total dosage of vasoactive. f. I² statistics indicate the possible presence of serious heterogeneity. g. The total number of patients evaluated for length of hospital stay was less than the total sample size included in this study. Ultimately, the number of patients assessed for hospital stay was 775 in the vitamin C group and 761 in the control group. h. I² statistics indicate the possible presence of milds heterogeneity. i. The total number of patients evaluated for length of ICU stay was less than the total sample size included in this study. Ultimately, the number of patients assessed for ICU stay was 813 in the vitamin C group and 791 in the control group. j. The statistical methods for urine output measurement were not specified in the studies. If urine output was based on patient self-reporting, it could introduce inaccuracies into the results.

Figure S1. Risk of bias assessment using the Cochrane RoB 2.0 tool.

Figure S2. The funnel plot for publication bias, comparing the overall mortality in the vitamin C group and control groups. A blue dot represents a single study, and the funnel dotted line represents 95% confidence intervals

Figure S3. Meta-analysis and forest plot of overall mortality. Very high dose vitamin C (≥12 g/day, or ≥150 mg/kg/day); high dose vitamin C (< 12 g, ≥6 g/day, or < 150 mg/kg/day, ≥75 mg/kg/day); low dose vitamin C (< 6 g/day, or < 75 mg/kg/day).

Figure S4. Meta-analysis and forest plot of overall mortality: the duration of vitamin C (＜3 days) and the duration of vitamin C (＞3 days).

Figure S5. Meta-analysis and forest plot of overall mortality: severe sepsis (SOFA≥10) and general sepsis (SOFA＜10).

Figure S6. Meta-analysis and forest plot of overall mortality: single center and multi center.

Figure S7 The change of vitamin C in plasma from baseline to post-treatment 48h or 72h. A. The change of vitamin C in vitamin C group. B. The change of vitamin C in control group

Figure S8. Meta-analysis and forest plot showed the length of hospital stay.

Figure S9. Meta-analysis and forest plot showed the length of ICU stay.

Figure S10. Meta-analysis and forest plot showed the urine output in the 24 h after treatment.
